# Immunoregulatory paracrine effect of mesenchymal stem cells and mechanism in the treatment of osteoarthritis

**DOI:** 10.3389/fcell.2024.1411507

**Published:** 2024-07-26

**Authors:** Xiuzhi Zhang, Tianhao Liu, Chunxiao Ran, Weidan Wang, Fengyuan Piao, Jiahui Yang, Simiao Tian, Lu Li, Dewei Zhao

**Affiliations:** ^1^ Department of Orthopedics, Affiliated Zhongshan Hospital of Dalian University, Dalian, Liaoning, China; ^2^ Orthopaedic Medical Research Center, Dalian University, Dalian, Liaoning, China

**Keywords:** osteoarthritis, mesenchymal stem cells, paracrine, cytokines, inflammatory response, immune regulation, repair of cartilage, exosomes

## Abstract

Osteoarthritis (OA) is a degenerative joint disease caused by chronic inflammation that damages articular cartilage. At present, the treatment of OA includes drug therapy to relieve symptoms and joint replacement therapy for advanced OA. However, these palliatives cannot truly block the progression of the disease from the immunological pathogenesis of OA. In recent years, bone marrow mesenchymal stem cell (BMSC) transplantation has shown great potential in tissue engineering repair. In addition, many studies have shown that BMSC paracrine signals play an important role in the treatment of OA through immune regulation and suppressing inflammation. At present, the mechanism of inflammation-induced OA and the use of BMSC transplantation in joint repair have been reviewed, but the mechanism and significance of BMSC paracrine signals in the treatment of OA have not been fully reviewed. Therefore, this article focused on the latest research progress on the paracrine effects of BMSCs in the treatment of OA and the related mechanisms by which BMSCs secrete cytokines to inhibit the inflammatory response, regulate immune balance, and promote cell proliferation and differentiation. In addition, the application potential of BMSC-Exos as a new type of cell-free therapy for OA is described. This review aimed to provide systematic theoretical support for the clinical application of BMSC transplantation in the treatment of OA.

## 1 Introduction

Osteoarthritis (OA) is a widespread joint disease worldwide. According to statistical data, OA affects approximately 10% of men and approximately 18% of women over 60 years old and significantly impairs quality of life. Consequently, OA has emerged as one of the main healthcare burdens in society (Glyn-Jones et al., 2015). The pathological characteristics of OA include articular cartilage degeneration, intra-articular inflammation with synovitis, and changes in subchondral bone (Chow and Chin, 2020). Due to the lack of vascular supply, innervation, and lymphatic reflux in cartilage, its self-healing ability is poor. Therefore, once articular cartilage undergoes degeneration or damage, it is highly susceptible to chronic inflammation (Abramoff and Caldera, 2020). Osteoarthritis is caused by immune imbalance in joint tissues, which can lead to degradation and damage of the surrounding cartilage, ultimately resulting in joint deformity and loss of function.

The main difficulties in the treatment of OA include a lack of radical treatment methods, irreversible disease progression, multiple factors affecting treatment effectiveness, and side effects of drug treatment (Chow and Chin, 2020). Compared with traditional treatment methods, stem cell transplantation has several advantages when treating OA, including the biological characteristics of BMSCs, such as inflammation regulation, multidirectional differentiation potential, and angiogenesis, which can eliminate chronic inflammation and promote cartilage repair (Shi et al., 2020; Zhou et al., 2023). Clinical trials of late-stage Kellgren Lawrence knee osteoarthritis showed significant improvements in OA symptoms 12 months after single transplantation of 50 million BMSCs, demonstrating the safety and effectiveness of BMSC transplantation (Chahal et al., 2019). Early views suggested that MSCs primarily promote OA repair by differentiating into chondrocytes. However, with the deepening of clinical research, it has been found that after a single injection of BMSCs for 6–12 months, there is no significant difference in the changes in cartilage defects, and the amount of cartilage does not significantly increase (Lee et al., 2019; Kim et al., 2023). However, compared with saline injection, a single MSC injection can significantly inhibit the disease progression of osteoarthritis, including significant improvement in VAS and WOMAC scores, and cessation of cartilage defect progression. This evidence strongly confirms that although MSCs have the potential for chondrocyte differentiation, their main mechanism of action in the treatment of osteoarthritis (OA) is to inhibit disease progression by regulating inflammation and immune responses, rather than relying on the chondrogenic differentiation ability of MSCs. In addition, studies have confirmed that after a single MSC transplantation for 3 years, the area of cartilage defects begins to show a downward trend, and there is no significant deterioration within 5 years after treatment (Kim et al., 2022). This indicates that in the short-term of treatment, MSCs do not show the ability to regenerate damaged cartilage, but long-term effects show that cartilage defects are partially repaired, possibly through self-repair after immune microenvironment recovery and/or MSC cartilage differentiation. Recently, further research on the paracrine effects of BMSCs has revealed their involvement in intercellular communication, the regulation of inflammatory mediators, the maintenance of immune balance, and the promotion of cell proliferation and differentiation through the secretion of soluble cytokines or exosomes. These secretions have beneficial effects and can alleviate inflammatory reactions and facilitate cartilage repair (Liu et al., 2020; Shao et al., 2022). Consequently, the paracrine effects of BMSCs hold significant importance in the treatment of osteoarthritis.

At present, many studies have shown that the paracrine secretion of mesenchymal stem cells participates in tissue repair and regeneration through immune regulation. For example, Bernardo et al. found that BMSCs induced the polarization from proinflammatory M1 macrophages to anti-inflammatory M2 macrophages through the production of prostaglandin E2 (PGE2) and indoleamine 2, 3-dioxygenase (IDO) and stimulated M2 macrophages to express interleukin-10 (IL-10), thereby alleviating OA and promoting cartilage regeneration (Bernardo and Fibbe, 2013). Liu Y confirmed that MSC-derived exosomes inhibited the release of inflammatory factors through the lncRNA-KLF3-AS1/miR-206/GIT1 axis, which promoted the proliferation of osteoarthritis chondrocytes and inhibited apoptosis (Liu et al., 2018a). Yang H. found that TSG-6 was secreted by bone marrow mesenchymal stem cells and inhibited the expression of inflammatory factors and matrix metalloproteinases by inhibiting the TLR2/NF-κB signalling pathway, which significantly improved the inflammatory microenvironment of the degenerative nucleus pulposus and reduced intervertebral disc degeneration (Yang et al., 2018). These studies suggested that the paracrine activity of BMSCs plays an important role in improving the immune microenvironment and promoting cartilage repair in OA.

The aim of this article was to provide a detailed and comprehensive theoretical basis, as well as new application insights, for the use of BMSC transplantation to treat osteoarthritis (OA) by comprehensively reviewing the mechanisms related to the paracrine effects of BMSCs on immune regulation and tissue repair.

## 2 The anti-inflammatory paracrine mechanism of BMSCs

At present, conservative treatment based on traditional drugs to alleviate pain is commonly used in for the clinic to treat OA, but this approach cannot achieve cartilage regeneration and repair (Cao et al., 2020). BMSCs can secrete anti-inflammatory factors such as interleukin-10 (IL-10), prostaglandin E2 (PGE2), transforming growth factor-β (TGF-β), and hepatocyte growth factor (HGF), thereby inhibiting the release of proinflammatory factors and exerting anti-inflammatory effects (Bernardo and Fibbe, 2013).

### 2.1 BMSC paracrine signalling inhibits the release of inflammatory factors

In OA, high levels of proinflammatory factors, such as TNF-α, IL-1β, and IL-6, are the main triggers of chondrocyte apoptosis, cartilage matrix degradation, cartilage collapse, and synovial infiltration. In addition, high levels of TNF-α can stimulate osteoclast differentiation and inhibit osteoblast function, leading to increased bone resorption (Osta et al., 2014). On the other hand, matrix metalloproteinase 13 (MMP-13) and thrombospondin motif-5 (ADAMTS5) can degrade cartilage extracellular matrix, which is composed of collagen and proteoglycans (Chow and Chin, 2020; Li T. et al., 2022). Proinflammatory factors such as TNF-α, IL-1β and IL-6 can stimulate the expression of MMP-13 and ADAMTS5 through the nuclear factor-κB (NF-κB), phosphatidylinositol 3-kinase/protein kinase B (PI3K/AKT) and mitogen-activated protein kinase (MAPK) pathways, thereby inducing the degradation of chondrocytes and matrix (Chow and Chin, 2020). Therefore, inhibiting the release of proinflammatory factors is a key strategy for the treatment of OA.

Available evidence has shown that BMSC transplantation significantly inhibits the upregulation of TNF-α, IL-1β, IL-6, and NF-κB p50 and p65 in OA, and OA symptoms are significantly alleviated (Hamdalla et al., 2022). NF-κB plays a key role in the release of proinflammatory factors. It is the main transcription factor of various proinflammatory factors and regulates the expression of proinflammatory factors such as TNF-α, IL-1β, IL-6, and MMP-13 (Foxwell et al., 1998). Abnormal activation of NF-κB is often observed in OA, suggesting that inhibiting NF-κB is an important strategy to block chondrocyte apoptosis and delay the progression of OA (de Andres et al., 2016; Li et al., 2019; Dong et al., 2022). BMSC-derived PGE2 is a paracrine cytokine that can inhibit NF-κB nuclear translocation and binding to DNA-binding sites, which inhibits the transcriptional activation of NF-κB and reduces the release of proinflammatory factors such as IL-1β and TNF-α (Gomez et al., 2005; Letourneau et al., 2011; Hu et al., 2017). Moreover, PGE2-induced chondrocytes inhibit the p38 MAPK signalling pathway by secreting IL-10, which inhibits the inflammatory response caused by neutrophil recruitment and activation during osteoarthritis (Ma et al., 2015; Ge et al., 2020).

In summary, blocking the transcriptional activity of NF-κB on inflammatory factors by paracrine factors produced by BMSCs is a key strategy to prevent the progression of OA.

### 2.2 BMSC paracrine anti-inflammatory factors

The anti-inflammatory factors secreted by BMSCs can prevent the occurrence and development of OA by reducing the destruction of chondrocytes by proinflammatory factors, thereby inhibiting chondrocyte apoptosis and the degradation of cartilage matrix. More importantly, High levels of inflammatory factors in the OA tissue microenvironment induce transplanted MSCs to release anti-inflammatory factors, thereby inhibiting the release of inflammatory factors by inflammatory immunocyt and reducing excessive inflammation (Francois et al., 2012; Noone et al., 2013; Chinnadurai et al., 2014; Croes et al., 2015; Noronha et al., 2019).

The anti-inflammatory protein TNF-α-stimulated gene 6 protein (TSG-6) is a 35–38 kD glycoprotein expressed by a variety of cell types in response to proinflammatory cytokines (Day and Milner, 2019; Yang et al., 2019). Previous studies have shown that bone marrow stromal cells can secrete TSG-6 to reduce the production of proinflammatory cytokines such as IL-1β, IL-6 and TNF-α by inhibiting activation of the TLR2/NF-κB signalling pathway, which improves the immune microenvironment (Choi et al., 2011; Liu et al., 2014a; Yang et al., 2020). In addition, MSCs release TSG-6 by inducing M1-to-M2 macrophage polarization, which inhibits the inflammatory environment and initiates tissue regeneration and repair (Song et al., 2018; Zhao et al., 2021). During TSG-6-mediated induction of macrophage phenotypic polarization towards the M2 type, the TLR4/MyD88/NF-κB pathway and TSG-6/CD-44 receptor pathways play key roles (Mittal et al., 2016; Song et al., 2017).

Interleukin-1 receptor antagonist (IL-1Ra) is a naturally occurring anti-inflammatory protein that can block the binding of IL-1α and IL-1β with their receptors and reduce the inflammatory response. Studies have shown that BMSCs can significantly block the production and/or activity of IL-1 and TNF-α in inflammatory tissues by paracrine IL-1Ra (Gabay et al., 2010; Zhao et al., 2013). In OA, BMSCs can secrete IL-1ra to prevent the interaction between IL-1α, IL-1β and their receptors, thereby inhibiting the destruction of cartilage by the release of TNF-α, MMPs and chemokines (Harrell et al., 2019a).

Indoleamine 2, 3-dioxygenase (IDO) is a molecular switch for the polarization of monocytes to M2 macrophages (Bernardo and Fibbe, 2013). In the inflammatory microenvironment, BMSCs secrete IDO to catalyse the degradation of tryptophan and form the immunomodulatory molecule kynurenine, which can change the microenvironment from immunogenic to tolerogenic. Moreover, in the presence of kynurenine, macrophages polarize to the M2 phenotype and secrete TGFβ or IL-10 to induce tissue regeneration and repair (Bianco et al., 2009; Ravishankar et al., 2012). Moreover, kynurenine produced by IDO induces the differentiation of helper CD4+ lymphocytes into regulatory T lymphocytes and directly inhibits cytotoxic CD8+ T cells, thereby inhibiting tissue inflammation (Munn and Mellor, 2016; Schafer et al., 2016). Studies have confirmed that IDO significantly inhibits inflammatory cell infiltration and cartilage destruction in arthritis, thereby alleviating inflammation and pain in OA (Criado et al., 2009; Partain et al., 2023).

## 3 The effect of BMSC-derived paracrine factors on the regulation of immune cells

Disturbances in the joint immune microenvironment can trigger pathological changes in OA, such as synovial inflammation, chondrocyte apoptosis, and excessive activation of immune cells (Dai et al., 2018). Recent studies have shown that BMSC transplantation significantly improves the hyperinflammatory state of various immune diseases, such as inflammatory bowel disease (IBD), graft versus host disease (GvHD), and autoimmune diseases (AIDs) (Al Jumah and Abumaree, 2012; Zhao et al., 2015; Canavan et al., 2016). This evidence suggests that bone marrow stromal stem cells have significant immunoregulatory functions and are potential immunoregulatory tools for repairing the immune microenvironment of OA.

### 3.1 The paracrine regulatory effect of BMSCs on macrophages

In OA, inflammatory factors such as TNF-α, IL-1β and IFN-γ are continuously produced and maintained at a high level. These inflammatory factors can stimulate joint tissue, leading to an increased inflammatory response, which in turn leads to a vicious cycle of more inflammatory factors being generated. Due to the influence of the inflammatory environment and the imbalance in the regulatory effect of cytokines on chondrocytes and stem cells, cartilage regeneration is weakened, and the inflammatory response is sustained and irreversible (Chow and Chin, 2020).

Within the innate immune system, it is well established that macrophages are key players that initiate and control inflammation (Mantovani et al., 2013). Proinflammatory macrophages (M1 polarization phenotype) play a leading role in the inflammatory response of OA. M1 macrophages produce a large number of proinflammatory factors, such as TNF-α, IL-12 and iNOS, to promote the continuation of the inflammatory response. Moreover, anti-inflammatory M2 macrophage secretion is inhibited, resulting in a weakened anti-inflammatory response. This imbalance in macrophage polarization is an important factor leading to a persistent irreversible inflammatory response (Liu B. et al., 2018). Phenotypic switching in M1 and M2 macrophages is critical for controlling inflammation, preventing excessive tissue damage, and initiating tissue repair processes (Nie et al., 2022).

When exposed to sufficient levels of proinflammatory cytokines such as TNF-α and IFN-γ, BMSCs induce M2 macrophage polarization by secreting soluble factors such as IDO and PGE2 to inhibit inflammation and promote tissue homeostasis (Bernardo and Fibbe, 2013). IL-10 produced by M2 macrophages can inhibit the expression of MMP-3, MMP-13 and nitric oxide synthase (iNOS) in chondrocytes, which promotes the synthesis of type II collagen and proteoglycan, thereby protecting cartilage and delaying joint degeneration (Liu et al., 2022). In addition, IL-10 can inhibit the synthesis and secretion of related proinflammatory cytokines such as IL-6 and TNF-α, thereby regulating inflammation. M2 macrophages also induce T cells to polarize into CD4+CD25+FoxP3+ regulatory T cells (Treg cells) by releasing chemokine ligand 18 (CCL18) to enhance the anti-inflammatory response (Bernardo and Fibbe, 2013). In summary, stem cell paracrine signalling regulates immune balance through macrophage phenotypic switching, which may be a key process in stem cell treatment of OA.

### 3.2 The paracrine regulatory effect of BMSCs on T cells

T cells are the major constituents of synovial infiltrates in the membranes of OA patients, and T-cell subsets such as Th1, Th17, and Treg cells have been found within synovial aggregates (Li et al., 2017). Th1 and Th17 cells are inflammatory effector T cells. Inflammatory factors such as Th1 cell-derived IL-2, IFN-γ, TNF-α, and Th17 cell-derived IL-17 are some of the main factors that cause early OA inflammation (Rosshirt et al., 2021). Treg cells are important immunoregulatory cells in many inflammatory and autoimmune diseases that secrete anti-inflammatory factors such as IL-10 and TGF-β to increase T-cell tolerance and slow the inflammatory response. The imbalance between the number and regulatory capacity of Treg cells and the Th1, Th17 cells is characteristic of the pathogenesis of OA (Li et al., 2017).

The immunomodulatory factors secreted by BMSCs exert immunosuppressive effects by regulating the functions of T cells, thereby controlling inflammation. In particular, BMSCs not only directly secrete TGF-β, PGE2, and soluble human leukocyte antigen-G (sHLA-G), but also indirectly induce M2 macrophages to secrete CCL18, which promotes the formation of CD4+CD25+FoxP3+ Treg cells (Selmani et al., 2008). Forthermore, Treg cells not only inhibit the proliferation of proinflammatory Th1 and Th17 cells but also secrete anti-inflammatory factors such as IL-10 and TGF-β (English et al., 2009). In addition, BMSCs directly secrete soluble factors (TGF-β, HGF, PGE2, IDO, etc.) to inhibit the formation of Th1 and Th17 cells (Harrell et al., 2020). Thus, BMSCs can restore the balance between inflammatory effector T cells and anti-inflammatory Treg cells.

### 3.3 The regulatory effect of BMSC-derived paracrine factors on dendritic cells

Dendritic cells (DCs) are heterogeneous antigen-presenting cells that play a crucial role in innate and adaptive immune responses. DCs are activated by dangerous molecular signals, upregulate costimulatory molecules, produce various cytokines and chemokines, absorb antigens and process them to provide antigens to CD8+ and CD4+ T cells (Balan et al., 2019). When the pattern recognition receptors (PRRs) on the surface of DCs bind to pathogen-associated molecular patterns (PAMPs) and damage-associated molecular patterns (DAMPs), the innate immune system is activated (Woodell-May and Sommerfeld, 2020). Studies have shown that abnormal hyaluronic acid and fibronectin are DAMPs that activate PRRs, such as TLR2 and TLR4, in DCs during OA, thereby activating the innate immune system. Once activated, the cells and proteases of the innate immune system act indiscriminately without regard to whether the attack is directed towards a contaminant or towards the self (Scanzello et al., 2008). In particular, conventional/myeloid DCs (cDCs) can activate Th1, Th2, Th17, and CD8+ T cells to secrete high levels of proinflammatory cytokines, leading to joint cartilage damage (Nedunchezhiyan et al., 2022). Because DCs play a key role in initiating immune responses, they are considered important targets for immunosuppression and inflammation control. Studies have confirmed that BMSCs not only inhibit the transformation of monocytes into immature DCs (IDCs) by secreting PGE2 but also inhibit the maturation of DCs by releasing TSG-6 protein to block the MAPK and NF-κB signalling pathways (Spaggiari et al., 2009; Liu et al., 2014b).

### 3.4 The regulatory effect of BMSC-derived paracrine factors on NK cells

Natural killer cells are innate cytotoxic lymphocytes that can kill virus-infected, aberrant, or transformed cells. In addition, NK cells can quickly release cytokines and growth factors, thereby exerting immunoregulatory effects and promoting inflammation. During synovial tissue infiltration of white blood cells in osteoarthritis, NK cells have been shown to be an important group that releases proinflammatory mediators and the inflammatory protease granzyme A to trigger cartilage damage and abnormal bone metabolism and repair (Benigni et al., 2017; Jaime et al., 2017). Spaggiari found that BMSCs could secrete prostaglandin E (PGE2) and indoleamine 2,3-dioxygenase (IDO) to inhibit NK cell proliferation, cytotoxicity and the release of inflammatory mediators, including IFN-γ, TNF-α, and GM-CSF (Spaggiari et al., 2008). In addition, paracrine factors derived from BMSCs can enhance the release of IL-10 from macrophages, which can upregulate the expression of suppressor of cytokine signalling 3 (SOCS3) by activating the JAK-STAT signalling pathway, thereby inhibiting the function and proliferation of NK cells (Liu et al., 2016).

## 4 The potential of BMSC-derived paracrine exosomes in the treatment of OA

Exosomes are small, secreted vesicles with lipid bilayers approximately 40–100 nm in size that are thought to primarily act as intercellular communication vehicles that transfer bioactive lipids, nucleic acids (mRNAs and microRNAs) and proteins to induce biological responses in recipient cells (Lai et al., 2015). It has been confirmed that BMSC-derived exosomes (BMSC-Exos) can inhibit pain and inflammation in the early stage of OA, reduce cartilage degeneration, and then promote cartilage matrix expression and subchondral bone reconstruction, thereby mediating joint recovery and regeneration (He et al., 2020; Zhou et al., 2020).

### 4.1 The anti-inflammatory mechanism of BMSC-Exos in OA treatment

The anti-inflammatory effect of BMSC-Exos depends on the regulatory characteristics of immune cells. MSCs can induce the formation of M2 macrophages, tolerogenic DCs and Treg cells through exosomes that deliver immunoregulatory miRNAs and immunoregulatory proteins, thereby inhibiting the inflammatory response mediated by M1 macrophages, dendritic cells, and CD4+ Th1 and Th17 cells (Harrell et al., 2019b). BMSC-Exos can promote the proliferation of Treg cells by effectively inhibiting the maturation of DCs, thereby exerting an immunosuppressive effect (Sang et al., 2023). In addition, several studies have confirmed that microRNAs in BMSC-Exos, such as miR-182 and miR-23a-3p, promote M1-to-M2 polarization in macrophages by targeting interferon regulatory factor 1 (IRF1), thereby inhibiting the inflammatory response and promoting tissue regeneration (Zhao et al., 2019; Li Z. et al., 2022). Moreover, MSC-Exo-derived lncRNAs, such as lncRNAMEG-3 and lncRNA-KLF3-AS1, inhibit IL-1β-induced senescence and apoptosis to maintain the chondrocyte phenotype by antagonizing IL-1β-induced induction of ADAMTS5 and MMP-13 in chondrocytes (Liu et al., 2018c; Jin et al., 2021).

### 4.2 The mechanism by which BMSC-Exos promote cartilage repair and regeneration

Furthermore, G-protein-coupled receptor kinase interacting protein-1 (GIT1) promotes chondrocyte proliferation and inhibits chondrocyte apoptosis, thereby maintaining joint homeostasis. However, in OA, an increase in miR-206 triggers cartilage degeneration by targeting the translation of GIT1 mRNA (Ni et al., 2018). Studies have confirmed that BMSC-Exo-derived lncRNA-KLF3-AS1 can competitively bind to miR-206, abrogating miR-206-mediated inhibition of the translation and expression of GIT1 and thereby inhibiting chondrocyte apoptosis and cartilage degeneration in an *in vivo* model of OA (Liu et al., 2018a). In addition, FoxO3 mediates the expression of Col II and aggrecan through Sox9, which protects cartilage and blocks the development of OA (Matsuzaki et al., 2018). It has been reported that lncRNA H19 delivered by MSC-Exos to chondrocytes could competitively bind to miR-29b-3p to relieve the repression of the target gene FoxO3, thereby enhancing chondrocyte migration and matrix synthesis and suppressing apoptosis and senescence (Yan et al., 2021). In summary, BMSC-Exo-derived signalling molecules, such as microRNAs and lncRNAs, are important mediators for chondrocyte repair and have a positive effect on the repair of OA.

## 5 Discussion and prospects

OA is a chronic degenerative disease characterized by degeneration and fibrosis in articular cartilage, subchondral bone sclerosis, synovial inflammation, joint swelling and pain, limited activity, and deformity. There are up to 300 million OA patients worldwide (Abramoff and Caldera, 2020). In the past, OA was considered simple joint wear, but now studies have shown that the interaction between immune cells and inflammatory factors plays an important role in the pathogenesis of OA. At present, the treatment methods for early- or mid-stage OA, such as nonsteroidal anti-inflammatory drugs, intraarticular injection of sodium hyaluronate, and arthroplasty, can only relieve symptoms and improve function but cannot block the progression of the disease (Freitag et al., 2016). OA patients with severe dysfunction can only choose surgical joint replacement, which may lead to postoperative complications such as deep vein thrombosis, joint infection, prosthesis loosening and fracture. Due to the limited service life of the joint prosthesis, young patients may face the risk of multiple revision surgeries, thereby increasing the patient’s medical expenses and physical and mental burdens. Therefore, there is an urgent clinical need for a treatment method that can reverse the progression of the disease during the early stage of OA to avoid patients being forced to choose joint replacement. BMSCs are thought to have a wide range of immunomodulatory properties that can change the levels of inflammatory factors by interacting with immune cells, thus exerting anti-inflammatory and cartilage protective effects (Singer and Caplan, 2011; Griffin et al., 2013). Therefore, BMSC therapy is a highly anticipated method for the prevention and treatment of osteoarthritis.

**FIGURE 1:**
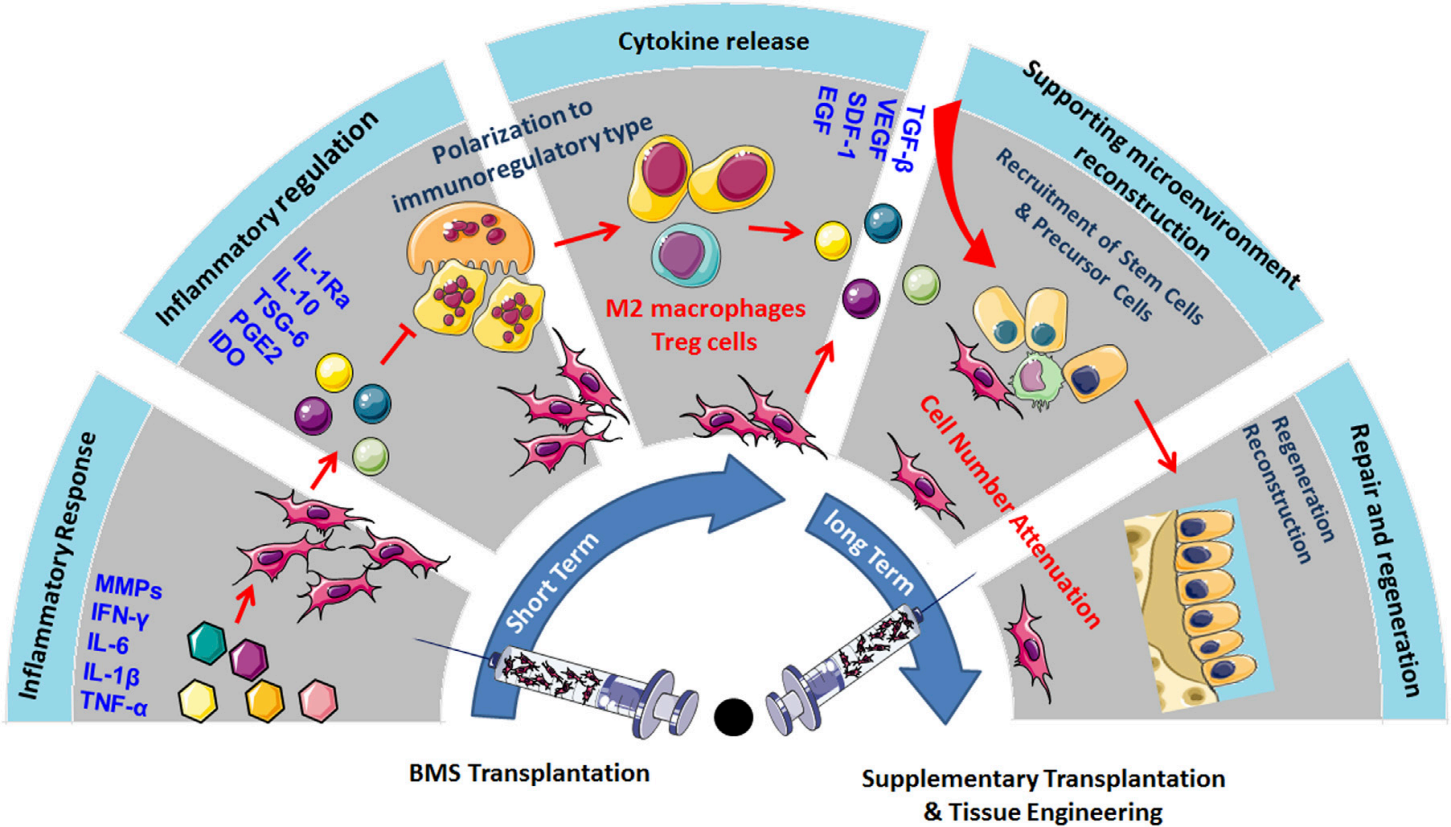
MSC transplantation could significantly inhibit chronic inflammation, alleviate pain, prevent the progression of OA, and show certain cartilage repair effects in long-term treatment. The primary mechanism lies in the immunomodulatory and anti-inflammatory properties of MSCs, which could create a regenerative microenvironment in damaged tissues, but the inflammatory and immune environment may limit their cartilage regeneration capabilities. Therefore, promoting MSC cartilage differentiation and regeneration through tissue engineering methods, considering factors such as cell quantity, source, injection timing, and frequency, could provide comprehensive support for OA treatment.

Existing clinical reports have shown that MSC transplantation can significantly inhibit chronic inflammation, alleviate pain, prevent OA progression, and demonstrate a certain cartilage repair effect in long-term treatment. The main mechanism is that MSCs have immunomodulatory and anti-inflammatory properties, which can establish a microenvironment promoting regeneration in damaged tissues (Colombini et al., 2019). Although the cartilage differentiation ability of MSCs is highly anticipated in OA treatment, but MSC transplantation has not shown ideal cartilage regeneration ability in the short term. This may be because the microenvironment in the body does not fully support the chondrogenic differentiation of MSCs. Although MSCs can differentiate into chondrocytes under specific culture media and growth factor stimulation *in vitro*, the complex inflammatory and immune environment *in vivo*, especially in the joints of OA patients, may limit the differentiation potential of MSCs (Peng et al., 2023). However, the inflammatory mediators produced by OA induce paracrine effects of MSCs, which also play an important role in immune regulation, recruitment of endogenous stem cells, and maintenance of the chondrocyte phenotype. Therefore, in the early stages of MSC transplantation, the cartilage differentiation rate of MSCs is not the main influencing factor. Only when the disease progression of OA is effectively suppressed can further promotion of MSC cartilage differentiation and regeneration through tissue engineering methods be considered, thus providing a comprehensive and effective OA treatment strategy. For example, using biodegradable scaffolds to provide 3D structural support for MSCs and promoting their differentiation into chondrocytes by loading specific growth factors such as TGF-β. Meanwhile, physical stimuli (such as mechanical stretching) and chemical stimuli (such as hypoxic conditions) can further enhance the cartilage differentiation efficiency of MSCs. Additionally, the cell number of MSCs, their cell source, the timing of injections, and the frequency of treatment are all critical factors in determining the chondrogenic differentiation of MSCs *in vivo* (Mamachan et al., 2024) (Figure 1).

At present, macrophages are considered key regulatory cells in OA-related inflammation. Targeting macrophages or changing their inflammatory phenotype is considered a promising strategy for the treatment of OA (van den Bosch, 2021). It has been reported that the dysregulation of macrophage conversion in the synovial membrane from the proinflammatory M1 subtype to the anti-inflammatory M2 subtype is an important cause of the occurrence and persistence of OA synovial inflammation (Xie et al., 2019). This chronic low-grade inflammation caused by the imbalance between M1 macrophages and M2 macrophages plays a key role in the pathogenesis and development of OA (Barreto et al., 2020). BMSCs can correct this immunological imbalance by secreting soluble factors such as IDO and PGE2 to induce the polarization of monocytes to M2 macrophages (Bernardo and Fibbe, 2013). Moreover, paracrine factors derived from BMSCs can promote the secretion of IL-10 by M2 macrophages, thereby inhibiting the NF-κB signalling pathway, inhibiting the production of inflammatory factors such as TNF-α, IL-1β and IL-6, and blocking the further development of OA inflammation (Letourneau et al., 2011). Therefore, intra-articular administration of MSCs to target inflammation and reduce the proinflammatory state of macrophages is a promising OA treatment strategy (van den Bosch, 2021).

In addition, BMSCs inhibit NK cell proliferation, cytotoxicity, and secretion of cytokines such as IFN-γ, TNF-α, and M-CSF by secreting PGE2 and IDO (Spaggiari et al., 2008). Cytokines such as IL-1ra, TGF-β, HGF, and PGE2 are secreted by BMSCs and can reduce the production of effector Th1 and Th17 cells. BMSCs can secrete TGF-β, PGE2, and sHLA-G5 to mediate the polarization of T cells into Treg cells (Selmani et al., 2008). These immunoregulatory properties of BMSCs have a positive effect on joint repair in OA, such as inhibiting excessive osteoclast differentiation, inducing the synthesis of cartilage type II collagen and proteoglycan, and inhibiting fibrosis and inflammation.

Mesenchymal stem cells have a naturally high degree of heterogeneity, and their factors include donor age, metabolic status, hormone level, genetic background and pathophysiological conditions. The heterogeneity of mesenchymal stem cells not only hinders the evaluation of the safety and efficacy of MSC therapy but also restricts the development of standardized and personalized MSC therapy (Kleineidam et al., 1997; McLeod and Mauck, 2017). For instance, the heterogeneity of mesenchymal stem cells is characterized by acquired cellular dysfunction in systemic diseases. In systemic diseases such as systemic lupus erythematosus, diabetes or rheumatoid arthritis, the therapeutic effect of allogeneic MSC transplantation is significantly better than that of autologous MSC transplantation (Costa et al., 2021). At present, autologous bone marrow- or adipose-derived MSCs are widely used to treat OA by intra-articular injection. Some clinical studies have shown that autologous MSCs from OA patients have an OA-related genetic background, such as abnormal expression of GDF5, which leads to a significant decrease in their ability to proliferate and differentiate into cartilage, thereby impairing their therapeutic effect (Wang et al., 2020). Because OA is more common in elderly patients with metabolic diseases, stem cell therapy for OA should focus on the heterogeneous factors of MSCs, including age, metabolic diseases, and genetic background (Vina and Kwoh, 2018). Among the many sources of MSCs, WJSCs have the advantages of homogeneity, including the most complete telomere length, the lowest immunogenicity, extensive differentiation potential, high proliferation ability and nontumorigenicity, and the extracellular matrix of WJSCs contains high levels of hyaluronic acid, sulfated glycosaminoglycan and collagen, which is highly similar to the extracellular matrix of chondrocytes. Therefore, by excluding the heterogeneity of the genetic background through gene sequencing, WJSCs may become the most suitable homogeneous MSC source for OA stem cell therapy (Matas et al., 2019).

MSC-derived exosomes have been shown to promote cartilage repair and regeneration in OA through different mechanisms, such as enhanced matrix synthesis, anti-inflammatory immune regulation, and the inhibition of apoptosis (Nguyen et al., 2021). In response to high levels of proinflammatory cytokines such as IFNγ, TNF-α, and IL-1β in the inflammatory environment, MSCs can release microRNAs such as miR-24-3p and miR-222-3p to drive macrophages from the M1 proinflammatory phenotype to the M2 proinflammatory phenotype and reduce matrix degradation (Domenis et al., 2018; Cheng et al., 2022). Therefore, engineered BMC-derived exosomes prepared by exposure to inflammatory factors or loaded with noncoding RNAs (including microRNAs, lncRNAs, and circular RNAs (circRNAs)) have been studied and used as cell-free treatments of osteoarthritis; however, these agents can not only overcome the uncertainty of cell heterogeneity in MSC treatment but have improved targeting for personalized OA treatment (Cheng et al., 2022).

In addition, The impact of disease tissue microenvironment on stem cell epigenetics is a current research hotspot in regenerative medicine (Falick Michaeli et al., 2022). Research indicates that the microenvironment of chronic tissue inflammation and tissue damage significantly affects the epigenetic state of MSCs, thereby initiating their tissue repair program (Jung et al., 2015). Epigenetic modifications include DNA methylation, histone modifications (methylation, acetylation), and non-coding RNA, which cause changes in gene expression levels without altering MSC sequences and are key pathways determining the fate of MSCs (Sharma and Bhonde, 2020; Wang et al., 2023). For example, inflammatory substances such as LPS in the inflammatory environment can induce MSCs to secrete more anti-inflammatory factors through histone methylation, thereby regulating the immune response and promoting tissue repair (Lin et al., 2019). These studies suggest that optimizing the epigenetics of MSCs through microenvironmental stimulation may have potential in the regeneration and repair of tissues affected by chronic inflammation (De Witte et al., 2018; Wang et al., 2023).

## 6 Conclusion

**FIGURE 2:**
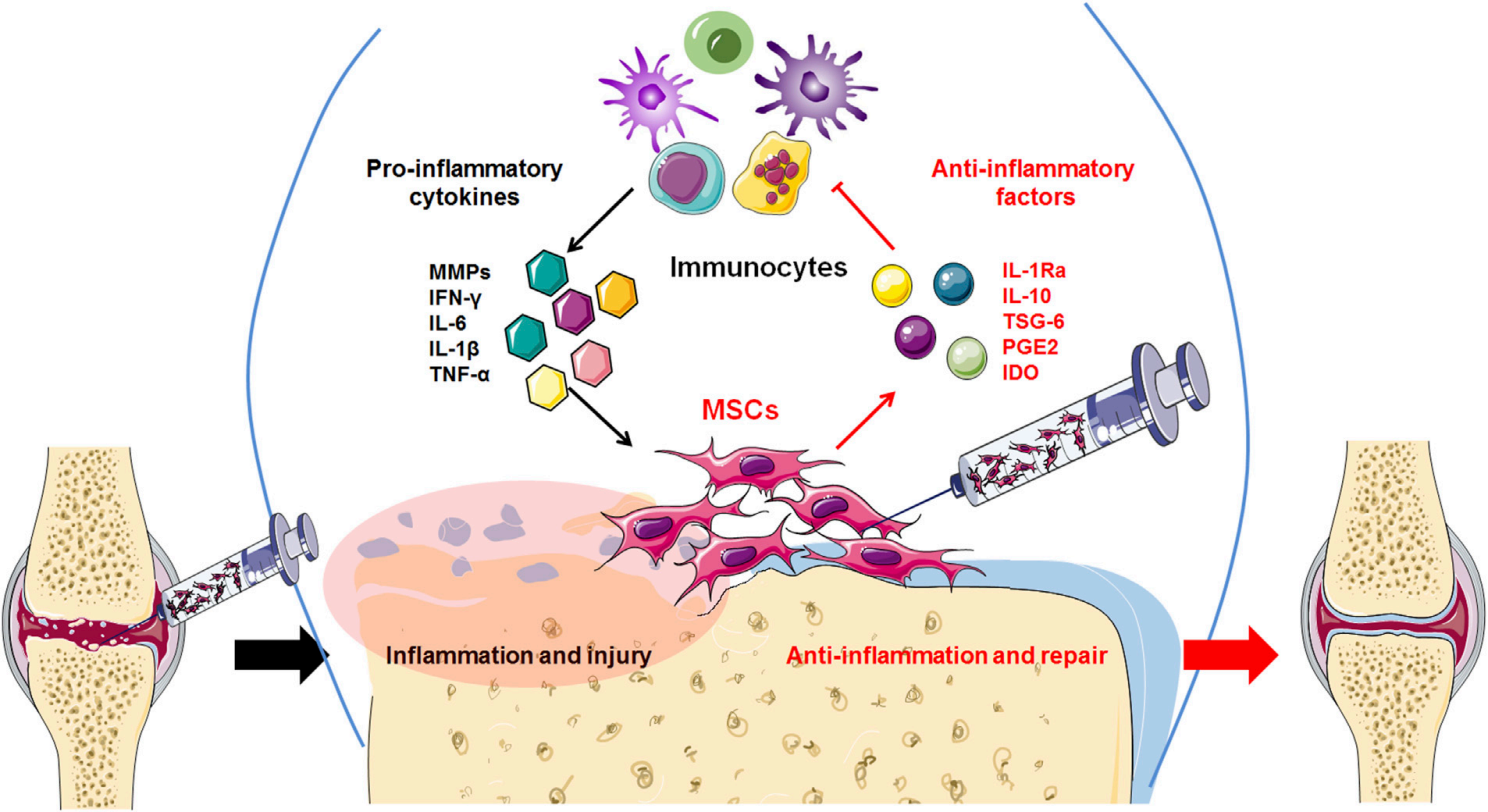
High levels of inflammatory factors in the OA tissue microenvironment induce transplanted MSCs to release anti-inflammatory factors, thereby inhibiting the release of inflammatory factors by inflammatory immunocytes and reducing excessive inflammation.

**FIGURE 3:**
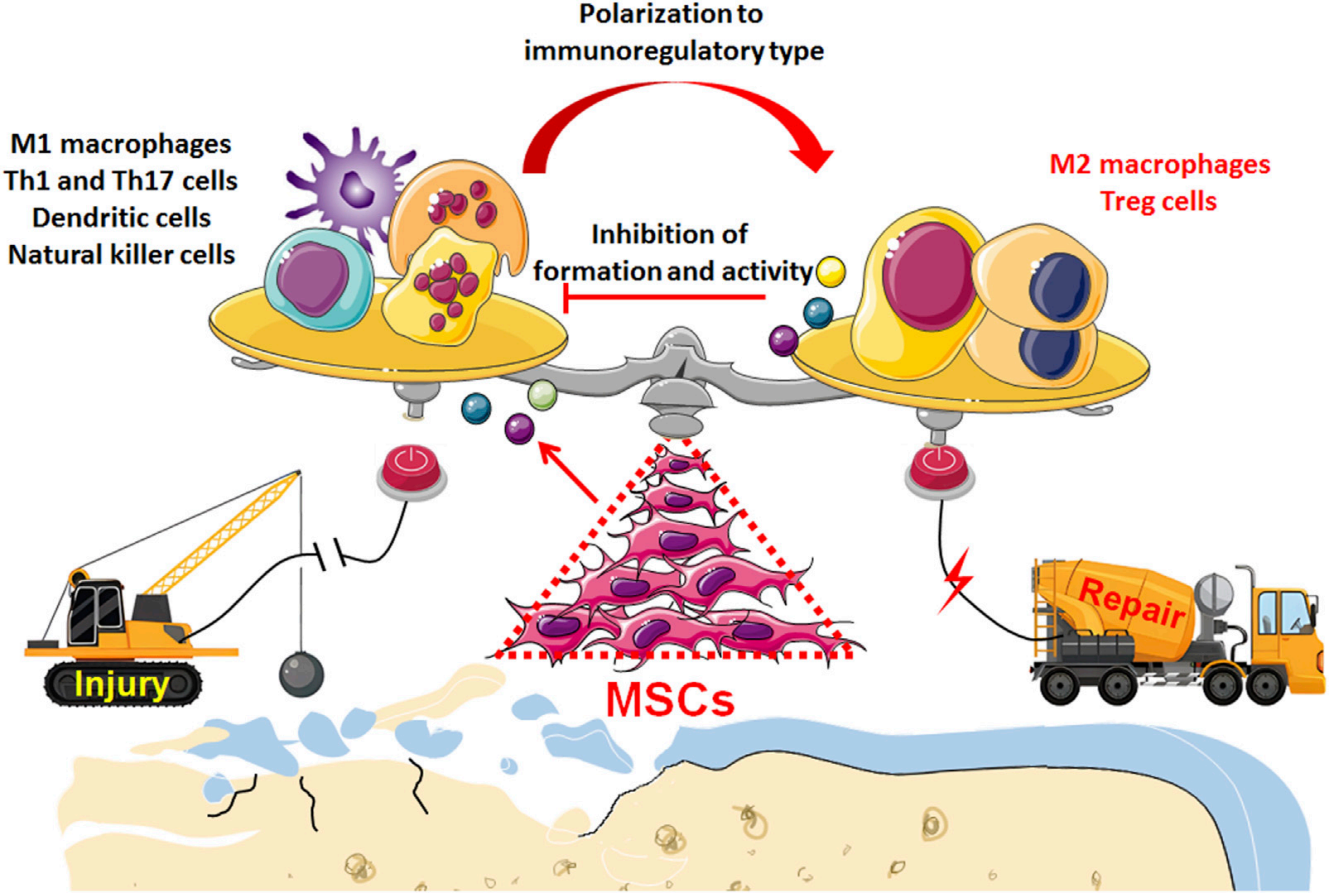
Transplantation of MSCs enables to restore the balance between inflammation and repair in the microenvironment of OA tissue: Through immunoregulatory paracrine, MSCs induce immunocytes to transform into anti-inflammatory and immunomodulatory phenotype such as M2 macrophages and Treg cells, which not only initiates the regeneration and repair of osteochondral tissue but also inhibits the excessive destruction of pro-inflammatory immunocyt.

Currently, osteoarthritis (OA) is not just joint wear and tear but a complex immunological disease. Previously, because of their ability to differentiate and regenerate cartilage, BMSCs were used to treat osteoarthritis through injection and transplantation, and significant therapeutic effects were observed. Current data indicate that cytokines and exosomes released by MSCs in response to inflammatory factors derived from OA play a major role in immunomodulation and establishing a microenvironment promoting regeneration (Figure 2). Moreover, the main therapeutic mechanism of MSCs is their regulatory effect on the inflammatory environment to restore immune and repair balance, such as by regulating the balance between Treg and Th17 T cells, as well as between M1 macrophages and M2 macrophages (Figure 3). These findings help to elucidate the role of MSC therapy and the mechanism, providing new inspiration for the development of MSCs to treat immune diseases and tissue engineering repair.
